# Reversing chromatin accessibility differences that distinguish homologous mitotic metaphase chromosomes

**DOI:** 10.1186/s13039-015-0159-y

**Published:** 2015-08-13

**Authors:** Wahab A. Khan, Peter K. Rogan, Joan H. M. Knoll

**Affiliations:** Department of Pathology and Laboratory Medicine, University of Western Ontario, London, N6A 5C1 ON Canada; Departments of Biochemistry, Computer Science, and Oncology, University of Western Ontario, London, N6A 5C1 ON Canada; Cytognomix, Inc., London, N6G 4X8 ON Canada

**Keywords:** Chromosome decondensation, Metaphase chromosome structure, Super-resolution microscopy, DNA Topoisomerases Type II, Fluorescence In Situ Hybridization, Epigenetics

## Abstract

**Background:**

Chromatin-modifying reagents that alter histone associating proteins, DNA conformation or its sequence are well established strategies for studying chromatin structure in interphase (G1, S, G2). Little is known about how these compounds act during metaphase. We assessed the effects of these reagents at genomic loci that show reproducible, non-random differences in accessibility to chromatin that distinguish homologous targets by single copy DNA probe fluorescence *in situ* hybridization (scFISH). By super-resolution 3-D structured illumination microscopy (3D-SIM) and other criteria, the differences correspond to ‘differential accessibility’ (DA) to these chromosomal regions. At these chromosomal loci, DA of the same homologous chromosome is stable and epigenetic hallmarks of less accessible interphase chromatin are present.

**Results:**

To understand the basis for DA, we investigate the impact of epigenetic modifiers on these allelic differences in chromatin accessibility between metaphase homologs in lymphoblastoid cell lines. Allelic differences in metaphase chromosome accessibility represent a stable chromatin mark on mitotic metaphase chromosomes. Inhibition of the topoisomerase IIα-DNA cleavage complex reversed DA. Inter-homolog probe fluorescence intensity ratios between chromosomes treated with ICRF-193 were significantly lower than untreated controls. 3D-SIM demonstrated that differences in hybridized probe volume and depth between allelic targets were equalized by this treatment. By contrast, DA was impervious to chromosome decondensation treatments targeting histone modifying enzymes, cytosine methylation, as well as in cells with regulatory defects in chromatid cohesion. These data altogether suggest that DA is a reflection of allelic differences in metaphase chromosome compaction, dictated by the localized catenation state of the chromosome, rather than by other epigenetic marks.

**Conclusions:**

Inhibition of the topoisomerase IIα-DNA cleavage complex mitigated DA by decreasing DNA superhelicity and axial metaphase chromosome condensation. This has potential implications for the mechanism of preservation of cellular phenotypes that enables the same chromatin structure to be correctly reestablished in progeny cells of the same tissue or individual.

**Electronic supplementary material:**

The online version of this article (doi:10.1186/s13039-015-0159-y) contains supplementary material, which is available to authorized users.

## Background

Large-scale chromatin reorganization from interphase to metaphase is driven by mitotic-specific condensation factors [1, 2]. Broadly speaking, this is thought to include histone proteins undergoing post translational modifications and interaction of histone tails with neighboring nucleosomes [1]. This is complemented with a network of non-histone proteins such as DNA methyltransferases involved in chromatin remodeling [3]. At later stages of the cell cycle, solenoidal supercoiling by topoisomerase concomitant with structural maintenance of chromosomal (SMC) proteins [4] further influences the condensation process.

Previous studies have used chromatin modifying reagents to study chromosome biology and investigate the large scale folding of the chromatin fiber. This has been performed, for instance, using chemical inhibitors which disrupt canonical chromatin-associating proteins [5–9] or enzymes which map chromatin accessibility in the human genome [10]. Our interest in chromatin accessibility arose out of an observation that short, locus-specific, single copy DNA probes detect differences in DNA compaction between homologs at ~10 % of allelic loci on mitotic metaphase chromosomes [11–13]. This is referred to as differential accessibility (or DA) to specific, condensed chromosomal targets. In human lymphocyte and lymphoblastoid cells, DA was non-random, heritable, and not unique to imprinted regions [13]. This led to the suggestion that DA represents an intergenerational mechanism of storing epigenetic information in mitotic metaphase chromosomes between parent and daughter cells [13].

The underlying basis for DA is not known. Here, we assess the contributions of different epigenetic factors towards these allelic differences in chromatin accessibility during metaphase. Cells are treated with chromatin-modifying reagents that are known to alter chromosome condensation, with the objective of providing insight into the basis of DA during mitotic metaphase.

## Results

### Effects of chromatin-modifying reagents on metaphase chromatin

Chromosome condensation was altered in two lymphoblastoid cell lines (GM06326, GM10958 obtained from NIGMS Cell Repository [Camden]) by separately treating them with several reagents, known to modify chromatin. Treatments were directed at essential DNA modifications, proteins altering DNA structure, and histone proteins with established roles in chromatin compaction and remodeling [1, 2, 8]. We assessed chromosome decatenation by inhibiting topoisomerase IIα with ICRF-193, histone dephosphorylation with okadaic acid (OA), histone deacetylation with trichostatin A (TSA), histone H3K27me3 demethylation with UNC1999, and DNA hypomethylation by incorporation of 5-azacytidine (5-AZC). We also analyzed metaphase chromosomes from cell lines of patients with cohesin mutations (Additional file 1: Table S1, GM20000 and GM20466).

**Fig. 1:**
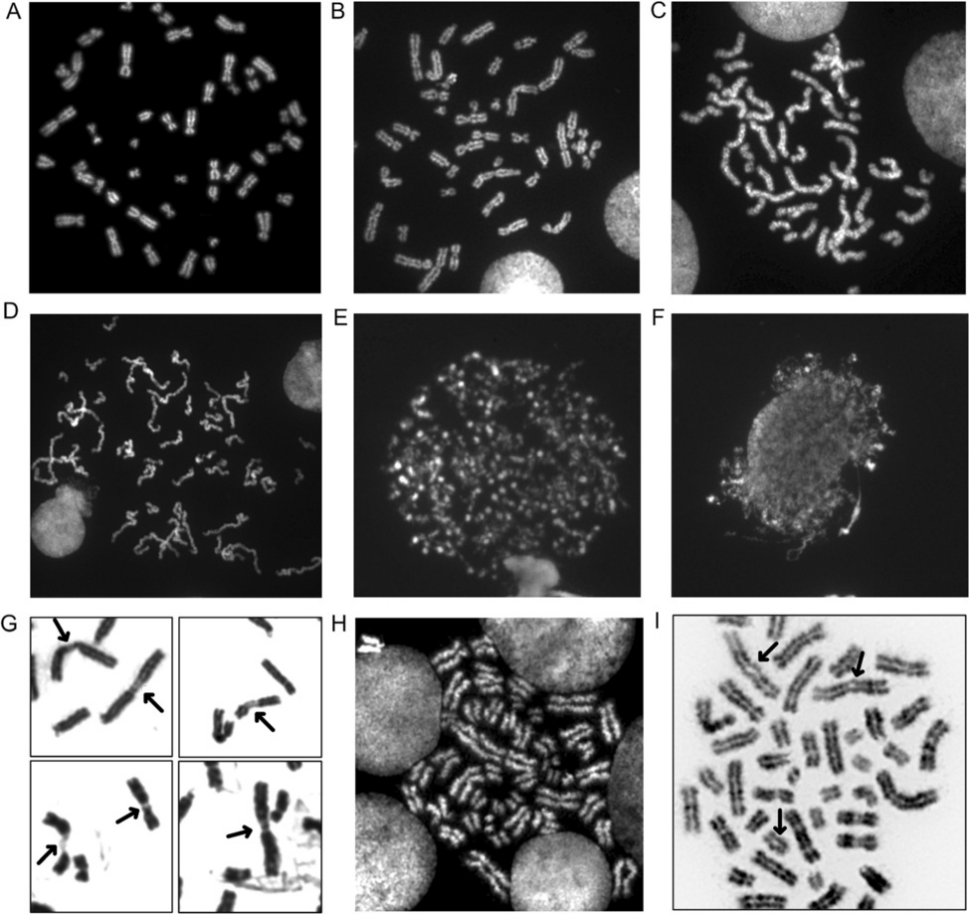
Decondensation treatments with visible effects on metaphase chromosome morphology. **a** Normal metaphase cell with no treatment. **b–d** ICRF-193 treated cells at increasing drug concentrations (0.10, 0.25 and 0.50 μM; left to right) show increasingly elongated chromosomes. OA treated cells with (**e**) early condensation at S and (**f**) late S phase of cell cycle. **g** 5-AZC treated metaphase chromosomes showing heterochromatin regions that did not condense (arrows). **h** Cell from individual with SC phocomelia (mutation in *ESCO2* c.604C > T, c.752delA, exon 3) showing premature sister chromatid separation primarily at heterochromatic regions near centromeres and (**i**) heterochromatic repulsion (arrows) in most pericentromic regions resulting in a railroad track appearance to the chromosomes. Metaphase chromosomes from Cornelia de Lange individual (*NIPBL* c.5721del5, exon 31) appeared similar to untreated normal cells (panel **a**)

Chromatin-modifying inhibitor concentrations were optimized in the cell lines to establish cytogenetic or immunofluorescence phenotypes in which the inhibitors’ effects were clearly detectable microscopically, without significantly compromising mitotic indices or chromosome identification. Compared to untreated controls (Fig. 1a), chromosome decatenation was decreased with 0.10–0.50 μM ICRF-193, which resulted in longer, entangled metaphase chromosomes (Fig. 1b-d, Additional file 2: Figure S1). Longer chromosomes (based on the International System for Human Cytogenetic Nomenclature, ISCN 2013 [14]) were apparent with increasing concentrations of ICRF-193. This was obvious at 0.25 and 0.5 μM concentrations, where there was a statistically significant shift in the 550 to < 700 band resolution category and the ≥ 700 band resolution category (*F* = 9.86, *p* = 0.0015). There was no significant difference in the 300 to < 400 and the 400 to < 550 band level categories (*F* = 1.93, *p* = 0.180) among untreated and ICRF-193 treated metaphases.

Incubation with 0.25 μM and 0.50 μM OA caused premature chromosome condensation (PCC) (Fig. 1e-f), as previously documented [5]. Inhibition of histone deacetylation and K27 trimethylation by TSA (at 0.40 μM and 15.0 μM) and UNC1999 (at 5.0 μM and 15.0 μM), respectively, produced metaphase chromosomes similar in morphology to untreated control metaphase chromosomes. For TSA (0.40 μM and 15.0 μM), a decrease in diploid mitotic cells (~1 % vs 5-6 % for untreated cells) and detection of occasional polyploid cells were also observed. For UNC1999 treatment, effects were confirmed by demonstrating substantially lower H3K27me3 immunofluorescence of interphase nuclei (Additional file 3: Figure S2). At the highest dose of UNC1999 [45 μM], absence of metaphase cells precluded further analysis. Incubation with 17.5 μM and 35.0 μM of 5-AZC showed decondensed heterochromatic regions (Fig. 1g), as previously reported [8]. At lower concentrations of 5-AZC (i.e. 3.5 μM and 7.0 μM), decondensation was not evident. As expected [15], immortalized cells from an individual with SC phocomelia showed absence of primary constriction (Fig. 1h) and/or heterochromatic repulsion (Fig. 1i) in chromosomes due to a cohesin mutation in *ESCO2* (Additional file 1: Table S1). Chromosomes of an individual with Cornelia de Lange Syndrome and a mutation in *NIPBL*, another cohesin gene, exhibited apparently normal morphology.

### Targeting topoisomerase IIα eliminates inter-homolog chromatin accessibility differences in metaphase at distinct loci with DA

In prior studies where we documented DA at ~ 10 % of the 305 genomic loci [11–13], ≥ 66 % of metaphase cells (two-proportion Z-test, *p* < 0.05) consistently exhibited non-random differences in DNA probe fluorescence intensity between homologous regions [13]. A set of single copy (sc) DNA probes for fluorescence *in situ* hybridization (scFISH), from imprinted and non-imprinted loci (*RGS7;* 2.09 kb, *CACNA1B*; 2.23 kb, *HERC2*; 1.82 kb, *SNRPN*; 2.08 kb, *ADORA2B*; 1.78 kb, *PMP22*:IVS3; 2.32 kb, *ACR*; 3.5 kb, see Additional file 1: Table S1 for genomic coordinates), were hybridized to metaphase chromosomes and scored for DA according to these criteria [13].

**Fig. 2:**
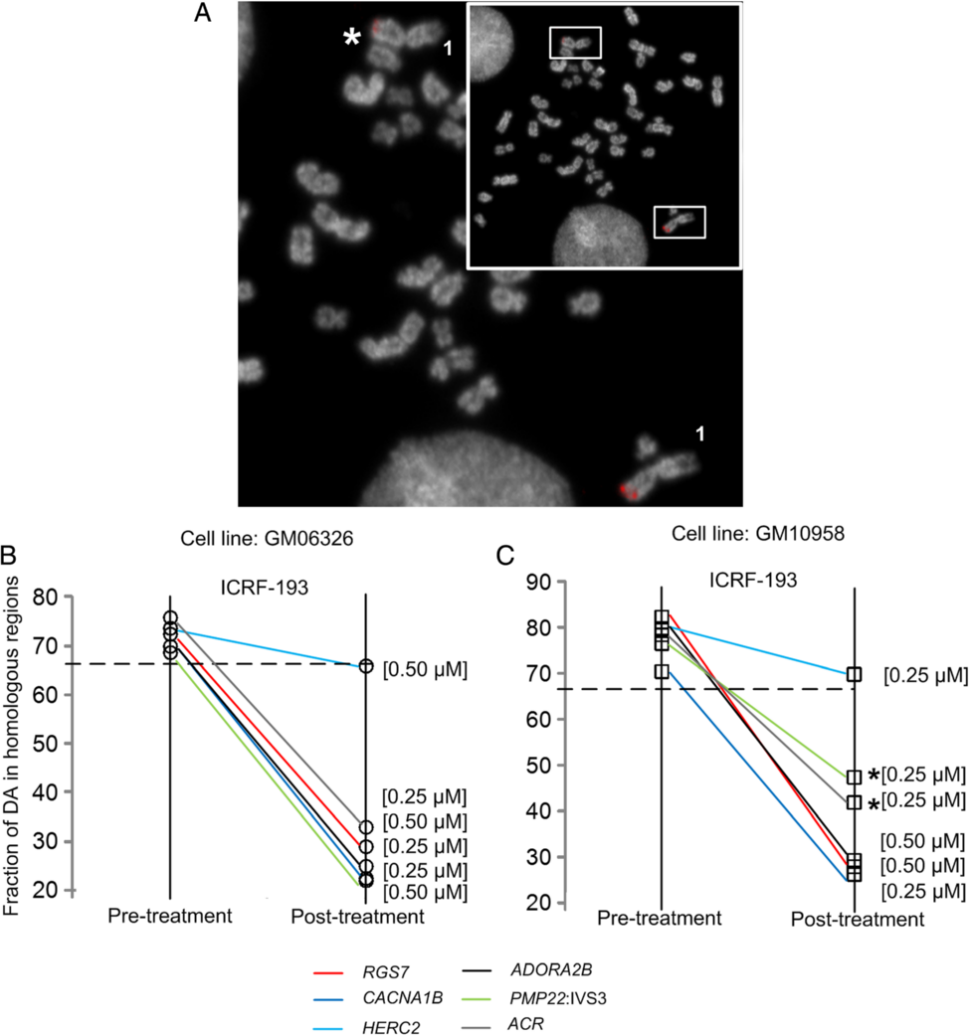
Representative example of differential accessibility (DA) and its reduction with topoisomerase IIα inhibitor ICRF-193. **a** Metaphase cell showing chromosome 1 homologs hybridized with single copy DNA FISH probe from within *RGS7* (2.09 kb). Relative to its homolog, * marks the chromosome with the weaker probe hybridization signal; indicating DA. Inset shows metaphase cell with homologs of interest (boxed). **b-c** Ladder plots compare effect of topoisomerase IIα inhibitor, ICRF-193, on DA at various concentrations and genomic loci in two lymphoblastoid cell lines. Colored lines connecting two points, pre and post-treatment (x axis), represent different genomic targets as indicated in the key. Frequency of DA to homologous regions is expressed as a percentage (y axis). Greater than two-thirds (dotted line) of the cells analyzed (*n* = 20–100 cells, *μ* = 43 cells/per target) in pre-treatment control showed DA. **b** In cell line GM06326, with the exception of *HERC2*, DA was significantly reduced post-treatment (z-score < −2.0, *p* < 0.05, two-proportion z test) at distinct genomic targets. **c** These findings were reproduced in a second cell line, GM10958, however in this case, reduction in DA was marginally significant at *PMP22*:IVS3 and *ACR* (indicated by *)

We examined the effects of modifiers of chromatin accessibility that alter DNA compaction (topoisomerase IIα) on DA (Fig. 2a). Inhibition of chromosome decatenation with the topoisomerase IIα inhibitor, ICRF-193, eliminated DA at multiple single copy loci, equalizing probe intensities on both homologs. This loss of DA was noted at multiple genomic targets in ICRF-193 treated cells (Fig. 2b-c), including *RGS7*, *CACNA1B*, *ADORA2B*, *PMP22*:IVS3, and *ACR*. It was related to a decrease in the amount of decatenation, resulting in reduced chromosome supercoiling. The effects of ICRF-193 on DA varied for certain genomic targets (e.g. *PMP22*:IVS3, *ACR*), between the cell lines (Fig. 2b-c). *HERC2* was the only exception of a locus that maintained differences in accessibility (DA) across a range of ICRF-193 concentrations (Fig. 2b-c, Additional file 1: Table S1). We suggest that the genomic context of this gene may explain the lack of response (see Discussion).

### Quantification of chromatin accessibility following topoisomerase IIα inhibition

**Fig. 3:**
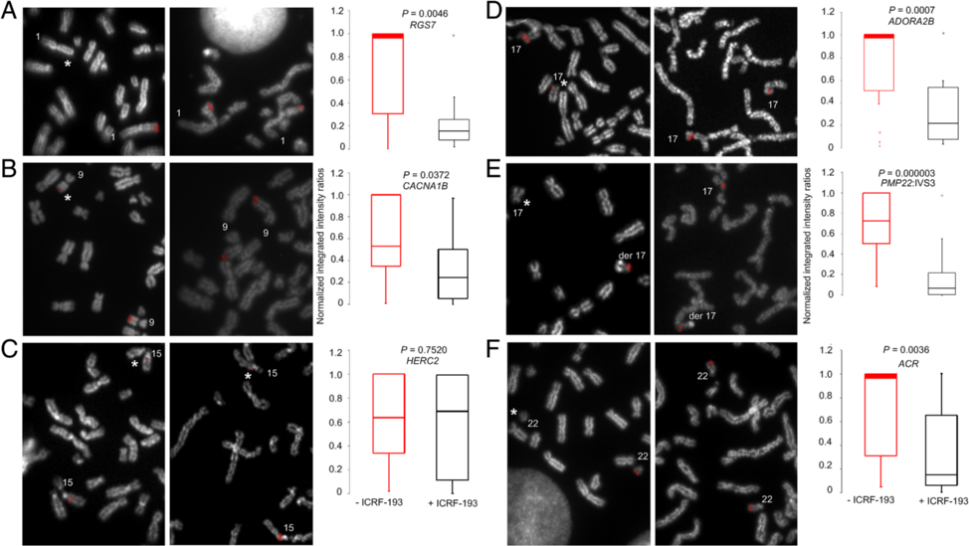
Quantification of inter-homolog fluorescence intensities following chromosome decondensation with ICRF-193. **a-f** FISH with single copy probes targeting six distinct genomic regions within chromosomes 1q43 (*RGS7*), 9q34.3 (*CACNA1B*), 15q13.1 (*HERC2*), 17p12 (*ADORA2B*, *PMP22*:IVS3), and 22q13.33 (*ACR*) are indicated. For untreated chromosomes (left column, panels **a-f**, respectively), probe signal is bright on one homolog and appears dim or not visible on corresponding target (*). For chromosomes treated with ICRF-193, (middle column, panels **a**-**f**, respectively) probe signal is bright on both homologs. Probes detecting DA exhibited larger differences in inter-homolog DNA probe fluorescence (red box plots in right column: median intensity ratios: from 0.53 to 1, *n* = 125 cells). ICRF-193-treated chromosomes exhibited smaller differences in DNA probe fluorescence (black box plots in right column: median intensity ratios from 0.08-0.27, *n* = 121 cells) (*p* < 0.05; two tail *t*-test), suggesting that both chromosomal homologs were equally accessible, except at the *HERC2* locus, where DA was not completely reversed. In instances where the median is coincident with the upper quartile, it is emphasized by a thick line to show distinction with median in corresponding category. The notation ‘der 17’ refers to a derivative chromosome 17 homolog resulting from a translocation between chromosome Y and 17

We quantified differences in probe hybridization between homologous loci using gradient vector flow (GVF) image analysis after ICRF-193 treatment, and compared results to untreated cells [13, 16] (Fig. 3, Additional file 4: Figure S3). Intensity differences in mean normalized probe fluorescence after ICRF-193 treatment were reduced by 2-fold (Δμ = 0.352) relative to untreated control cells (Δμ = 0.725) for *RGS7*, *CACNA1B*, *ADORA2B, PMP22*:IVS3, and *ACR* (Fig. 3, Additional file 4: Figure S3), indicating that the drug equalizes accessibility of the probe to both homologous targets. In contrast, the intensities of a probe detecting DA within *HERC2* were similar in treated (Δμ = 0.662) and untreated cells (Δμ = 0.713) (Fig. 3c, Additional file 4: Figure S3C).

**Fig. 4:**
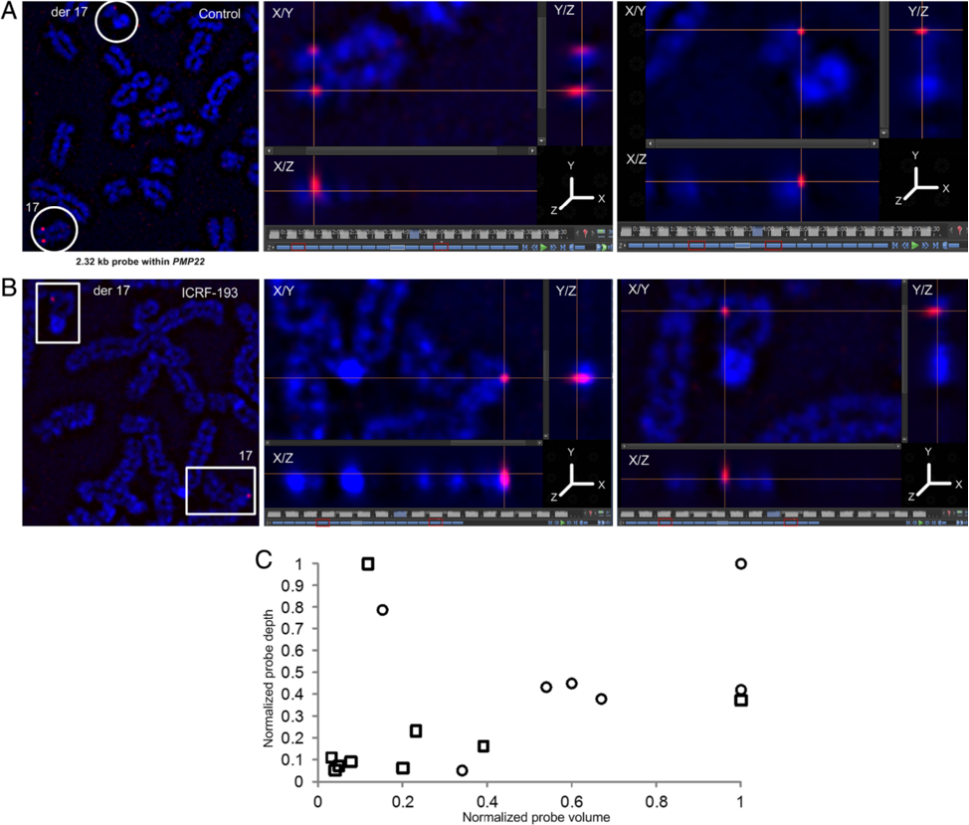
Visualization of internal chromosome accessibility with super resolution 3D-SIM. **a** Untreated metaphase cell showing DA between chromosome 17 homologs (left panel, circled) hybridized with single copy FISH probe within *PMP22*:IVS3 (2.32 kb). Probe depth spans 1.30 μm or 10 of 17 (middle panel, red boxes*) and 0.65 μm or 5 of 17 (right panel, red boxes*) optical sections within accessible and less accessible homologs, respectively. **b** Decondensed metaphase chromosomes (left panel, boxed) hybridized with same *PMP22*:IVS3 (2.32 kb) single copy probe exhibit equal accessibility to both homologs. Probe depth (10 of 17 and 11 of 17 sections) for each homolog spans 1.30 μm (middle panel) and 1.43 μm (right panel), respectively. Same cell line (GM06326) is used in (**a**) and (**b**). Crosshairs are over maximal fluorescence. Der 17 refers to derivative chromosome 17. This was used as a cytogenetic marker to distinguish parental homologs. **c** Scatterplot of individual cells showing differences in hybridized probe volume and depth for untreated and treated cells. Normalized mean differences in hybridized probe volume (Δμ = 0.730 μm^3^, circles) and depth (Δμ = 0.651 μm, squares) for different untreated cells (*n* = 10 cells) for genomic target (*PMP22*:IVS3) with DA. These were significantly greater (volume: *p* = 0.003, depth: *p* = 0.013; two-tailed *t* test) compared to the same genomic target post-treatment (indicated with squares) in which both alleles were accessible (volume: Δμ = 0.237 μm^3^, depth: Δμ = 0.238 μm, *n* = 9 cells). Single cell outliers (y_max_ or x_max_) with ICRF-193 treatment did not affect p-value cut off (*α* = 0.05). Normalized probe volume and depth were not strongly correlated pre- (*r* = 0.559) and post-treatment (*r* = 0.164). *Left red box is position zero for all panels in (**a**) and (**b**)

Super-resolution, 3-dimensional structured illumination microscopy (3D-SIM) provided direct evidence of the effects of ICRF-193 on equalization of chromosome target accessibility. 3D-SIM increases the spatial resolution with which metaphase chromatin accessibility can be visualized and quantified. Larger volumes and greater depths of probe hybridization are consistent with decreased condensation and lower DNA superhelicity. Quantification of the volumes occupied by the hybridized probe showed large differences in the distributions of probe depth between homologs in untreated cells with DA (for example, *PMP22*:IVS3; Fig. 4a). By contrast, Fig. 4b shows the effects of ICRF-193 treatment with the same probe, notably that both chromosomes are hybridized to similar depths and occupy equivalent volumes, consistent with abrogation of DA. Overall, probe volumes and depths were consistently different between untreated and treated categories (Fig. 4c). The differences in probe hybridization volume are also visualized with 3D-anaglyph displays of untreated (Additional file 5: Movie S1) and ICRF-193-treated (Additional file 6: Movie S2) chromosome homologs from the same metaphase cells.

### Inhibitors of histone modifications, cytosine methylation, and mutations in cohesin, a non-histone protein, do not alter DA

**Fig. 5:**
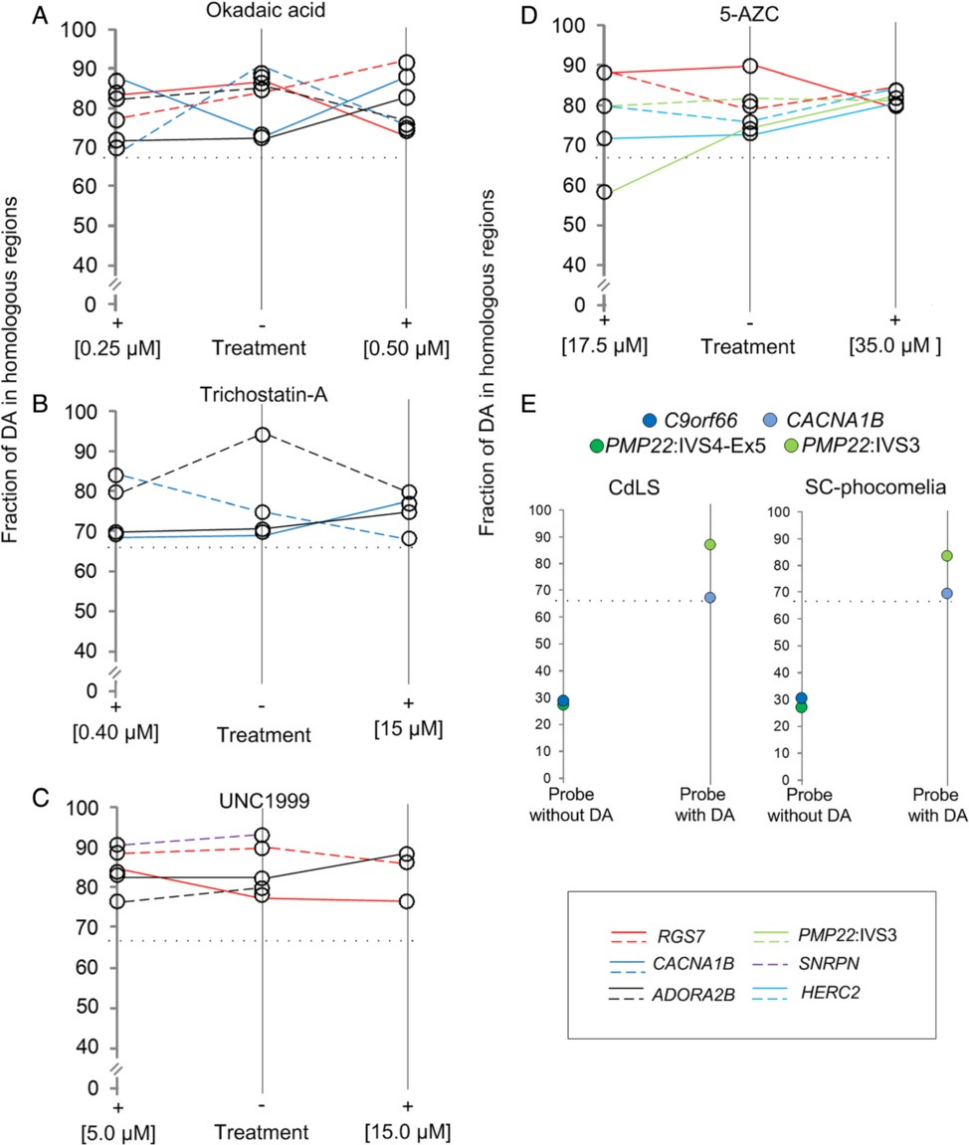
Pre- and post-treatment effects of chromatin-modifying reagents and cells with cohesin mutations on DA. **a–e**. Ladder plots compare fraction of DA (i.e. expressed as a percentage along y axis) with (+) and without (−) chromatin-modifying reagents at various concentrations (x axis). Fraction of DA is illustrated with solid and dashed lines for GM06326 and GM10958 cells, respectively. Each line color corresponds to a different probe (indicated in key; *RGS7*, *CACNA1B*, *ADORA2B*, *PMP22*:IVS3, *SNRPN*, *HERC2)* or control probes exhibiting equal accessibility (*C9orf66*, *PMP22*:IVS4-Ex5). **a-c** In all cases, greater than two-thirds of the cells analyzed (*n* = 20–100, *μ* = 43 cells/per target) maintained DA pre- and post- reagent treatment in both cell lines at all concentrations tested. Black dotted line indicates threshold for DA. This suggests allelic chromatin accessibility differences were not reversed with chromatin-modifying reagents targeting histone proteins. This was also true for chromatin-modifying reagents that prevent (panel **d**) cytosine methylation or cohesin mutations (panel **e**) in cells from individuals with Cornelia de Lange Syndrome (CdLS) and SC-phocomelia Syndrome. Probes that do not detect DA, *C9orf66* and *PMP22*:IVS4-Ex5, were hybridized to cell lines with cohesin mutations, as controls. Outlier in panel D (*PMP22*:IVS3) refers to ~ 60 % of the cells (*n* = 41 cells total) with DA in cell line GM06326 following 5-AZC [17.5 μM]

We also examined the effects of histone modifications that typically influence interphase chromatin accessibility on DA in metaphase. Differences in probe hybridization intensity were unperturbed by treatment with either OA or TSA (Fig. 5a-b, Additional file 1: Table S1). Besides prematurely condensed diploid cells, OA also produced a rare population of prematurely condensed tetraploid-like cells [due to unscheduled DNA replication [17]*,* in which the extra pair of homologs did not hybridize using probes from within distinct genomic targets (*ADORA2B* with DA or *PMP22*:IVS-Ex5 no DA, Additional file 7: Figure S4). This suggests that these cells have to complete mitosis in order to re-establish their respective allelic accessibility patterns. Inhibition of H3K27me3, a characteristic of transcriptionally repressed chromatin [7] and reported to be distributed across metaphase chromosomes [18], by UNC1999 also had no effect on DA (Fig. 5c, Additional file 1: Table S1). For all probes and both cell lines, at least 66 % of metaphase cells (*n* = 20–100 cells, *μ* = 43 cells/per genomic target) retained differences in fluorescence intensities for each probe (Fig. 5a-c, Additional file 1: Table S1). Also, DA was not altered by loss of DNA methylation (Fig. 5d, Additional file 1: Table S1) or mutations in two different cohesin genes (Fig. 5e, Additional file 1: Table S1).

## Discussion

In this study, we investigated epigenetic modifications responsible for allelic differences in chromatin accessibility reported between homologous mitotic metaphase chromosomes [13]. Our results demonstrate that accessibility differences between allelic loci on metaphase chromatin can be equalized by inhibition of topoisomerase IIα, which controls levels of DNA superhelicity during condensation [19], and do not reflect underlying histone modifications [20], regional decompaction by cohesin mutations [21, 22], or effects of deoxycytosine methylation [8].

ICRF-193 attenuates variation in epifluorescent probe signal intensities from specific loci that exhibited DA (Fig. 2, Fig. 3 and Additional file 4: Figure S3) and this attenuation was further confirmed by quantifying hybridized probe depth and volume using super-resolution 3D-SIM (Fig. 4). ICRF-193 is a bisdioxopiperazine compound that disrupts the catalytic activity of ATP-bound DNA topoisomerase IIα, rendering the enzyme inactive and preventing DNA decatenation [19, 23]. ICRF-193 was selected as there was evidence for its ability to affect chromatin condensation in mitotic metaphase without causing cell death [9]. We recognize, however, that the catalytic activity of topoisomerase IIα is required at multiple steps of decatenation (including DNA binding, cleavage or strand passage); however, it is not certain where in the topoisomerase reaction cycle [19, 23], DA is attenuated.

ICRF-193 has also been used to produce decondensed metaphase chromosomes for high resolution chromosome analysis [9]. The feasibility of this approach to reduce condensation of mitotic chromosomes in order to increase chromosome length, DNA accessibility, or alter gene expression has previously been demonstrated [24–26]. Different inhibitors of topoisomerase IIα [19] can prevent DNA binding by topoisomerase IIα, compete with ATP (simocyclinone d8), block ATPase activity (novo- and cholorobiocins) or are irreversible, effectively poisoning the enzyme (etoposides) [19]. Assays for DA that alter chromatin condensation should avoid those with high cytotoxic and genotoxic effects, such as doxorubicin.

Attenuation of DA required a specific and sustained effect on metaphase chromosome decompaction, without loss of chromosome integrity. Inhibiting the catalytic activity of topoisomerase IIα changes the overall morphology of mitotic chromosomes (Fig. 1b–d, Additional file 2: Figure S1) by altering levels of axial condensation, leading to extended, catenated metaphase chromosomes [9]. ICRF-193 specifically hinders compaction of 300-nm chromatin fibers to form into chromatids with prometaphase-level compaction [27]. Since ICRF-193 targets the early stages (prophase, pre-metaphase) of mitotic chromosome condensation [27], DA seems most likely to become established in early metaphase. Inhibiting or disrupting metaphase chromosome compaction and reversal of DA likely depends on the stage of chromosome condensation at which the inhibitor acts. Our findings are consistent with the possibility that by changing DNA topology, less accessible DNA targets on one homolog become more accessible. Distinct levels of DNA catenation of each homolog could be established, for example, through differences in the local concentration of topoisomerase IIα bound to metaphase chromosomes [24], structural differences between homologs that impact substrate accessibility at the target chromosome loci or a combination of both.

Topoisomerase IIα is rapidly degraded as the cell enters G1. This is followed by a rise in its expression at G2/M, which is greatest among proliferating cells [28]. An increase in log phase growth or expression of topoisomerase IIα lowers sensitivity to topoisomerase inhibitors [29]. Thus, the degree to which endogenous topoisomerase IIα is inhibited by ICRF-193 in culture is likely to vary. This is relevant since loss of DA, while evident in both cell lines (e.g. GM06326, GM10958), did not occur to the same degree at the *PMP22*:IVS3 and *ACR* loci (Fig. 2b-c). The genomic target within *HERC2* notably showed similar percentages of DA in ICRF-193 treated and untreated cells (Fig. 2b-c). One possible explanation for this is the presence of extremely long palindromes (~210 kb), adjacent to and including *HERC2* segmental duplications [30], that might result in structural configurations that are simply recalcitrant to hybridization [31] or experimentally-induced chromosome decompaction.

Changes in chromatin accessibility have been associated with post-translational modifications to histones [20]. While we did not observe an effect from histone modifying enzymes, DNA modifications (DNMT1) or cohesin mutations on reversing DA (Fig. 5), it remains possible they could contribute to the observed allelic structural differences. In particular, histone modifications tend to be dynamic, are active at earlier points in the cell cycle, and often have antagonistic [20] effects on chromatin structure [6, 17, 20], which could mask their impact on DA. For example, restoration of expression of an inactive allele [32] coincides with the loss of trimethylated lysine in histones [33, 34]. It is conceivable that multiple histone modifications may need to be targeted to trigger an effect on DNA accessibility at higher levels of chromatin organization [20]. Currently, there is little evidence that these modifications are relevant to metaphase chromatin accessibility, have a sustained effect on the higher order chromatin folding, or are even present on mitotic chromosomes [35].

**Fig. 6:**
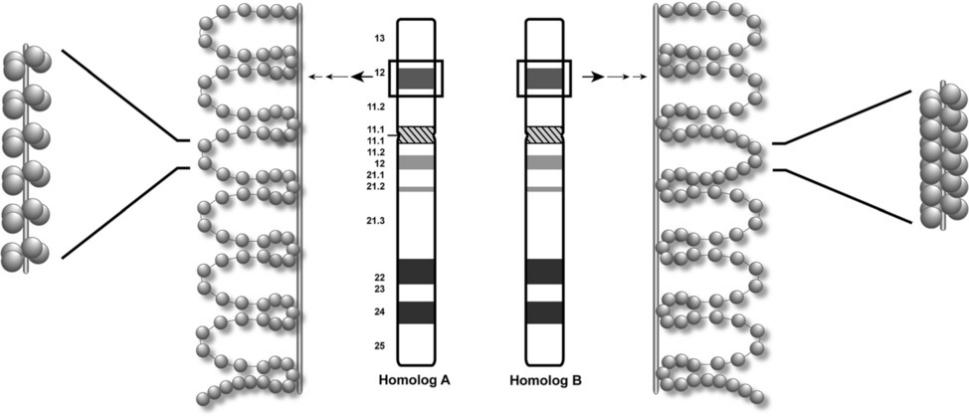
Working model of solenoidal supercoiling between homologous regions with differential accessibility. The model illustrates localized differences in chromatin accessibility at specific homologous loci in untreated cells (i.e. with DA, default state). Example of a homologous chromosomal region within 17p12 (black rectangle) ideogram is shown in the middle of the illustration. Immediately flanking each ideogram, chromatin loop size, its frequency, and distance between each helical turn (i.e. helical pitch) is kept the same for both homologs but inter-homolog accessibility within a localized loop is depicted to be variable. For homologous region A, this is illustrated as low-level compaction with widely spaced gray circles in contrast with high-level compaction in homologous region B. The outer most images show a partial cross section of each loop. In homolog A, the solenoid structure with greater accessibility has low longitudinal supercoiling vs. homolog B. For simplicity, additional levels of packing beyond the 300 nm loop fiber (indicated by multiple black arrows) are not shown. Chromatin loops are drawn in two-dimensions of a 3-D configuration found *in vivo*

The majority of cells with DA show quantifiable non-random differences in accessibility between homologous regions, typically expressed as a fraction of cells in a given individual [13]. The near absence of marks of open interphase chromatin, moreover, from DA regions [13] may confer differences in the chromatin state at the end of interphase that affect the density, binding, or activity of topoisomerase IIα to each allele. Our current results imply that inhibition of topoisomerase IIα reverses DA and restores equivalent accessibility by preventing disparities in superhelicity between homologous regions (Fig. 6). Otherwise, lax structural regulation by topoisomerase IIα in DA regions would enable these differences to persist. One possible mechanism to explain this might be that the regions where DA is observed in a fraction of cells are not as highly structurally regulated as loci displaying equivalent accessibility to both homologs. Conversely, this hypothesis suggests that the structural state of equivalently accessible regions maintains a strict degree of regulation, dictated in part by an array of open chromatin marks established during the prior interphase, as previously demonstrated [13].

Given the above considerations on formation of DA or equivalent accessible loci through separate topological constraints on chromatin, it still remains to be determined what benefit, if any, such differences would have in the cell. Our view is that DA is a structural feature that distinguishes homologous regions. Highly supercoiled regions with decreased accessibility alternate with more accessible domains along a chromosome. Decondensation at the end of mitosis could be driven by unwinding in these regions.

In interphase, homologous chromosomes are in repulsion relative to heterologous pairs [36]. Regions with DA may also spatially organize homologous regions in the mitotic nucleus. Such a mechanism could prevent allelic regions from being co-localized [36, 37] at or near DA loci, whereas equivalently accessible loci could be spatially clustered in the nucleus [38, 39].

Chromatin and transcriptional patterns of genes detected by single copy probes do not support a role for DA in regulation of expression. mRNA levels of these genes are very low or undetectable (Additional file 8: Table S2). Autosomal monoallelic expression [37] is therefore unlikely to be an effect of DA. Further, histone marks of transcriptionally active chromatin (H3K36me3, H4K20me1) do not significantly differ between DA and equivalently accessible loci (Additional file 8: Table S2). Finally, topoisomerase inhibitors have inconsistent effects on gene expression in DA regions. Topoisomerase poisons, such as camptothecin, which primarily inhibit topoisomerase I activity but also partially effect topoisomerase II, have been shown to downregulate *RGS7* (1.8 fold reduction), *SNRPN* (3.1 fold reduction), *HERC2* (2.2 fold reduction), *PMP22:*IVS4-Ex5 (1.6 fold reduction), *PMP22*:IVS3 (1.6 fold reduction), and *ACR* (1.38 fold reduction), but upregulate *CACNA1B* and *ADORA2B* by 1.21 and 1.44 fold, respectively [40]. Given these findings, it is possible that the observed differences in mitotic chromatin accessibility are not sufficient to dictate changes in gene expression.

DA is the result of differential activity by topoisomerase IIα at specific loci and particular homologous chromosomes. From chromosome conformation capture data [35], others have suggested that the formation of DA or equivalent accessible loci occurs *de novo* in every cell. This seems unlikely in light of the results reported here and in our previous study demonstrating that DA was transmitted from parental to progeny cells, and between related individuals [13].

## Conclusions

In conclusion, we show that DA is a stable structural mark of metaphase chromosomes that was not influenced by inhibitors of histone-modification, cytosine methylation or cohesin mutations that alter chromosome accessibility during interphase. Nevertheless, inhibition of topoisomerase IIα can reverse DA. We demonstrate that targeting the catalytic activity of ATP-bound DNA topoisomerase IIα, equalizes superhelical densities between metaphase chromosome homologs in locus-specific regions of DA. This raises the possibility that in normal untreated cells, the winding number of topoisomerase-induced, solenoidal supercoils can vary between homologous sequences within these regions (Fig. 6). Combined with our previous study [13], this suggests that DA is the result of variable catenation levels at specific loci which are distinguishable and heritable between homologous chromosomes.

## Methods

### Cell line and single copy DNA probe selection

Human lymphoblastoid cell lines were obtained from NIGMS Human Genetic Cell Repository [Coriell Institute, Camden, New Jersey]. These cell lines were selected based on microscopically visible chromosome translocations of known parental origins in which DA probes were hybridized. The translocations were used to mark cytogenetically distinguishable parental homologs in order to determine whether or not DA occurred randomly between homologs. This approach obviated the need to use other methods such as parental origin determination (POD-FISH) [41] to discriminate homologs. The characterization of DA on cell lines used in the present study (GM06326, GM10958) has been previously determined by single copy DNA FISH probes [13]. The homologous targets detected by these FISH probes are chromosomally normal. Additional cell lines with mutations of core cohesin components (Coriell Institute; GM20000, GM20466; Additional file 1: Table S1), causing chromatin decompaction [21], were also tested as potential indicators for DA. Single copy FISH probes detecting no DA (i.e. equivalent accessibility) (*C9orf66, PMP22*:IVS–Ex4) [13] were used as control hybridizations in cells with cohesin mutations alongside DA probes (*CACNA1B*, *PMP22*:IVS3). All cells were cultured, harvested for metaphase chromosomes, and processed for single copy FISH as described previously [42, 43]. Lymphoblastoid cells were cultured in RPMI-1640 medium (Gibco, Life Technologies Inc. ON, Canada) supplemented with L-glutamine, 15 % fetal bovine serum (Gibco) and 1 % penicillin/streptomycin (Gibco). Chromosomes were harvested in logarithmic growth by arresting cells in metaphase [10 μg/ml colcemid, Gibco) followed by incubating in 0.075 M KCl hypotonic solution and subsequently fixing the cells in 3:1 methanol:acetic solution. Single copy probes detecting DA were tested on different concentrations of decondensation treatments in each cell line (Additional file 1: Table S1). Probes were selected from within chromosomal regions representative of telomeric, pericentromeric and loci adjacent to these sites. Details by which single copy probes are designed, developed and used to analyse differences in chromatin accessibility has been described elsewhere [11–13, 31].

Genomic locations of single copy intervals were determined computationally [31], and they can be developed from any unique region in the genome (e.g. exons, introns, intergenic). Unlike BAC, cosmid or fosmid probes, single copy probes do not contain high copy repetitive elements. They were amplified using long PCR from genomic DNA and labelled by nick translation with biotin-dUTP (Roche Diagnostics, ON, Canada) or digoxigenin-dUTP (Roche Diagnostics, ON, Canada). Labelled single copy probes were detected with Cy™3 conjugated to IgG fraction monoclonal mouse anti-digoxin (Jackson ImmunoResearch, PA, USA) (diluted 1:200 [1.7 mg/ml]) or Alexa Fluor® 488 conjugated to streptavidin (Jackson ImmunoResearch, PA, USA) (diluted 1:500 [1.5 mg/ml]) [11–13] .

### Chromatin decondensation treatments

Chromatin-modifying reagents were incorporated *in vitro* into rapidly dividing, nonsynchronized lymphoblastoid cell cultures. The reagents targeting non-histone proteins included ICRF-193 (a bisdioxopiperazine derivative inhibitor of mammalian DNA topoisomerase IIα; Sigma-Aldrich) and 5-AZC (inhibits DNA methyltransferase; Sigma-Aldrich). Condensin mutations were not studied because they result in a loss of chromosome structural integrity and mislocalization of topoisomerase IIα [44]. Targets of histone proteins included OA (inhibitor of protein phosphatase I and IIα; Sigma-Aldrich), TSA (inhibitor of histone deacetylase; Sigma-Aldrich), and UNC1999 (small molecule inhibitor of histone lysine methyltransferases EZH2 and EZH1 catalyzing H3K27me3; Sigma-Aldrich). Each treatment dose (Additional file 1: Table S1) was optimized to our experimental design using baseline concentrations previously reported from pharmacokinetic, biochemical, and cytological studies on lymphocyte, HeLa or MCF-7 cells [5–9]. This was important, as it minimized cell toxicity and preserved chromosome morphology and banding for homolog identification following FISH. Specifically, final concentrations in cell culture ranged from 0.05–3 μM (ICRF-193), 0.1–0.5 μM (OA), 0.2–15 μM (TSA), 5–45 μM (UNC1999), and 3.5–35 μM (5-AZC). Using published time points as a baseline [5–9], duration in cell culture was 0.5, 1, 20, 72, and 7 h for ICRF-193, OA, TSA, UNC1999 and 5-AZC, respectively. Changes to higher order chromatin structure were visualized by DAPI-staining and epifluorescence microscopy before performing metaphase FISH. Untreated control cell cultures (i.e. no decondensation treatments) were taken through the chromosome harvest and FISH procedures simultaneously with treated cells.

For ICRF-193 treated and untreated cultures, 50 metaphases from each concentration (0, 0.1 μM, 0.25 μM, 0.50 μM) were assessed for chromosome resolution according to ISCN 2013 [14]. Dark bands were counted on at least two of chromosome regions 6p, 17q, 18q, 15q12/q24qter, or 22q in the cell lines. These chromosomal regions were selected because they are informative at most ISCN band resolution levels [14] and could be readily identified amongst the entangled, catenated chromosomes. Differences in chromosome resolution with varying ICRF-193 treatments were analyzed for significance (*α* = 0.05, one-way ANOVA test).

### Immunofluorescence

Immunofluorescence staining of nuclear histone protein H3K27me3 was achieved with a rabbit IgG monoclonal antibody to H3K27me3 according to the manufacturer’s protocol (Cell Signaling Technologies). This was performed to determine whether UNC1999 had an effect in reducing H3K27me3. Briefly, human lymphoblastoid cells were fixed in methanol/water (50:50 vol/vol), immediately spun onto microscope slides using a cytospin microcytocentrifuge (Statspin®), immersed in blocking buffer (0.3 % triton X-100 with 3 % BSA in 1X PBS) for 1 h, and incubated with a primary rabbit monoclonal antibody against H3K27me3 overnight at 4 °C (antibody diluted in same diluent as blocking buffer except with 1 % BSA). Cells were washed in 1X PBS and detected with goat anti-rabbit IgG conjugated to Dylight® 488 fluorochrome (Abcam®) for 1 h at 37 °C, followed by three 5 min washes in 1X PBS, and counterstained with DAPI. Nuclei were examined for presence of punctuate granular fluorescent signals. All UNC1999-treated cell cultures were set-up in duplicate. One set was harvested for metaphase chromosomes to evaluate the level of DA and the corresponding culture set was processed for immunofluorescence staining of nuclei, as described above.

### Quantification of DA following metaphase chromosome decondensation

Single copy FISH probe hybridization analysis was performed on Zeiss AxioImager.Z2 epifluorescence microscope and cells imaged with a CoolCube 1 camera using Metafer software (Metasystems). With background corrected, integrated probe signal intensities were determined using our previously described gradient vector flow (GVF) algorithm [13, 16]. GVF outlines the boundary of scFISH probe signals by computing a binary edge map from the gray scale image. From this active contour, the integrated intensity values (in pixels) were automatically computed for probe signal on each homolog in MATLAB, and a GVF result of the binary contour provided as an output. Probe signal intensities were then normalized by taking the difference in integrated intensities between homologs, and dividing by the sum of the intensities of both homologs in a given cell.

Using 3D-SIM (Nikon Corporation), inter-homolog probe volume and depth were also quantified in treated cells relative to untreated controls. 3D-SIM images were reconstructed with NIS-Elements AR software (version 4.13.00, Nikon Canada Inc.) as previously described [13]. The lateral fluorescence depth of a probe’s signal on a given homolog was calculated from reconstructed optical sections. Reconstructed optical sections were generated by taking the 2D layers of a captured image (each layer corresponding to an optical slice) and superimposing each layer into a 3D projection using Z-stack ND module within NIS-Elements. Each section was collected in 0.13 μm steps from a total of 17 reconstructed optical sections. The reconstructed image of the metaphase chromosome was then displayed in 3D volume view. The Nikon Elements Movie Maker option in ‘Directors Mode’ was subsequently used to add key frames in order to define the initial zoom, position, and rotation of the 3D metaphase chromosome in object space. The final movie was created by interpolation between these key frames. Predefined rotation presets were implemented which combined a 360° turn around the X/Y/Z-axis, while building up the image with additional sections along the Z plane. Volume of probe fluorescence was calculated following image segmentation and thresholding. All parameters quantified were analyzed for significance (*α* = 0.05, two-tailed *t* test).

### Internal hybridization controls

BAC probes (obtained from The Center for Applied Genomics, Toronto), were co-hybridized with scFISH probes, as positive hybridization controls and to identify the homologs of interest in ICRF-193 treated samples. BAC probes were labelled with Spectrum Green-dUTP (Abbott Molecular) and were located on chromosome 9p21.2 and 11q12.2, spanning 187 kb (RP11-57P14) and 188 kb (RP11-467L20), respectively. DA was not observed using BAC probes. For example, the mean normalized probe fluorescence intensity differences between homologs for a BAC probe mapping to chromosome 9p21.2 (RP11-57P14), that was co-hybridized with the scFISH probe from *CACNA1B*, was Δμ = 0.147 [0.25 μM ICRF-193] and Δμ = 0.156 [0.10 μM ICRF-193] (Additional file 9: Figure S5A-B, Additional file 10: Table S3). Similarly BAC probe RP11-467L20, that was co-hybridized with the scFISH probe from *RGS7*, exhibited mean normalized probe fluorescence intensity differences of Δμ = 0.104 [at 0.50 μM ICRF-193] and Δμ = 0.162 [at 0.25 μM] (Additional file 9: Figure S5C-D, Additional file 10: Table S3). Inter-homolog chromatin compaction differences detected with scFISH probes showing DA, were not evident with cohybridized BAC probes (Additional file 9: Figure S5).

### Evaluating Copy Number Variations (CNVs) in regions with DA

Genomic locations of single copy probes were evaluated relative to locations of common CNVs detected in two normal control populations whose samples have been tested with high resolution Affymetrix Cytoscan HD copy number and SNP microarray platform. CNV datasets were derived from the Ontario Population Genomics (OPGP) Platform (~895 individuals of European ancestry, at least 25 probes per CNV [45]) and Healthy sample group (HS; ~400 individuals, at least 35 probes per CNV; obtained from Affymetrix). Single copy probes utilized in this study did not overlap any normal population CNVs (Additional file 8: Table S2). CNVs, therefore, do not account for differences in probe hybridization intensities between homologous chromosomes [13].

Additionally, none of the single copy probe targets reported in the current study were localized close to the CNVs in GM06326 (Additional file 8: Table S2). CNVs in cell line GM06326, initially characterized using Affymetrix Genome-Wide Human SNP Array 6.0 [46], were analysed in our laboratory using the Affymetrix Chromosome Analysis Suite (ChAS). The intensity data file (.CEL) from this cell line was converted into genotyping data files using the Affymetrix Genotyping Console Software. The resulting copy number CHP data file (.CNCHP) was visualized using Affymetrix NetAffx (v. 32.1) library files with Affymetrix ChAS software (high resolution setting: 100 kb gain or loss, at least 50 probes per CNV). In GM06326, CNVs (>100 kb; [GRCh37]) were observed on 7 chromosomes. They include: 1q43 (243,078,262 - 243,303,154; gain), 2p11.2 (89,144,034-89,399,953; gain), 8p23.2 (3,899,581-4,283,153; gain), 8p11.22 (39,256,048-39,386,953; loss), 15q11.2 (21,104,604-22,317,726; loss), 16p11.2 (32,113,669-32,630,542, loss; 34,527,248-34,765,204, gain), 19p12 (21,256,875-21,393,137; gain), and Xq21.31 (90,765,370-90,998,761; gain).

## Additional files

Additional file 1: Table S1. Human lymphoblastoid cell lines harvested for metaphase chromosomes were hybridized with single copy (sc) probes from indicated GRCh37 genomic coordinates. Concentrations of each treatment, optimized for *in situ* hybridization are indicated. Only cells treated with 5 μM of UNC1999 from GM10958 cell line were included in the statistical analysis of DA for *SNRPN* and *ADORA2B*, due to an insufficient number of mitoses at 15 μM. The numbers of metaphase cells with and without (w/o) DA are indicated for each treatment dose along with corresponding no treatment control prepared at the same time. Additional file 2: Figure S1. White box indicates zoom in view of entangled or catenated metaphase chromosomes following **(A)** 0.25 μM and **(B-C)** 0.50 μM ICRF-193 treatment. Reduced supercoiling is visible as cytologically unwound chromatids (*). **Panel B** is same cell as shown in Fig. 1d. Additional file 3: Figure S2. Immunofluorescence staining of lymphoblastoid nuclei following selective inhibition of H3K27me3 associated with inactive chromatin. **(A)** H3K27me3 staining shows bright punctate nuclear signals in untreated cells, but diminished fluorescence and reduced signals post-treatment with **(B)** 5 μM and **(C)** 15 μM UNC1999, respectively. Additional file 4: Figure S3. Quantification of inter-homolog probefluorescence intensities following chromosome decondensation with ICRF-193 in independent cell lines. **(A–F)** Box plots show normalized integrated intensity ratios (y axis) following scFISH for six distinct genomic regions within chromosomes 1q43 (*RGS7*), 9q34.3 (*CACNA1B*), 15q13.1 (*HERC2*), 17p12 (*ADORA2B*, *PMP22*:IVS3), and 22q13.33 (*ACR*) in untreated (−) and treated (+) cells (x axis). GVF measurements in cells hybridized with single copy probes detecting DA from within *RGS7*, *HERC2*, *PMP22:*IVS3, and *ACR* are indicated from cell line GM10958. Measurements of normalized inter-homolog intensities for *CACNA1B* and *ADORA2B* are indicated from cell line GM06326. The same genomic regions were hybridized in opposite cell lines and inter-homolog differences quantified as shown in Fig. 3. Probes detecting DA exhibited larger differences in inter-homolog DNA probe fluorescence (red box plots: median intensity ratios: from 0.68 to 1, *n* = 125 cells). ICRF-193 treated chromosomes exhibited smaller differences in DNA probe fluorescence (black box plots: median intensity ratios from 0.15-0.39, *n* = 118 cells) (*p* < 0.05; two tail *t*-test), suggesting retrieval of the less accessible chromosome target, except in the case of *HERC2*, in which DA was not completely reversed. In instances where the median is coincident with the upper quartile, it is emphasized by a thick line to show distinction from the median in the corresponding category. Additional file 5: Movie S1. 3D anaglyph view of single copy FISH target (*PMP22*:IVS3) between chromosome homologs. Chromosome 17 homologs appear in object space rotated 360° around the z axis at 15 frames per second to emphasize DNA probe volume in context of reconstructed chromosomes. The parental homologs were distinguishable based on a Y;17 chromosome translocation in cell line GM06326, resulting in a normal chromosome 17 and a derivative (der) 17. Probe volume inside the metaphase chromosome in left panel (corresponding to normal chromosome 17 in Fig. 4a) exhibits greater occupancy compared to its less accessible target (right panel, corresponding to der 17 in Fig. 4a), depicting inter-homolog DA from all perspectives. Reconstructed optical sections were taken over 17 z-stacks, at 0.13 μm per stack, with 3D-Structured Illumination Microscopy. Additional file 6: Movie S2. 3D anaglyph view of single copy FISH target (*PMP22:*IVS3) between chromosome homologs following topoisomerase IIα inhibition. Chromosome 17 homologs appear in object space rotated 360° around the z axis at 15 frames per second to emphasize DNA probe volume in context of reconstructed chromosomes. Probe volume inside the metaphase chromosome in left panel (corresponds to normal chromosome 17 in Fig. 4b) is similar compared to the other homologous target (right panel, corresponds to der 17 in Fig. 4b) depicting equalization of DA from all angles. Bottom panels show still image of equalized probe fluorescence without chromosome context. Reconstructed optical sections were taken over 17 z-stacks, at 0.13 μm per stack, with3D-Structured Illumination Microscopy. Additional file 7: Figure S4. Examples of DA in tetraploid-like cells after okadaic acid treatment. **(A)** Chromosome 17s are marked with centromeric probe (D17Z1, green) to identify the four copies in the tetraploid-like cell (boxed). **(B)** Tetraploid-like cell shows two of the four homologs hybridized with a 1.78 kb scFISH probe (red) within *ADORA2B* on chromosome 17p12, indicating DA. **(C)** Zoom in view of the same cell from panel B shows a bright hybridization to the normal chromosome 17 and a weaker hybridization to its corresponding homolog (observed in *n* = 16/25 cells). **(D)** The other pairs of normal chromosome 17 and der 17 (asterisk) showed absence of hybridization to their respective allelic targets (*n* = 12/28 cells). The same outcome shown in **panel D** was predominantly observed in a region with no *DA (PMP22:* IVS-Ex5*)* in which two of the four homolog pairs did not hybridize (*n* = 13/17 cells). Tetraploid-like cells with only single chromosome hybridizations, hybridizations to only two normal chromosome 17s or two der 17s were excluded, as they could not be analyzed for DA which is assessed between homologs. Additional file 8: Table S2. CNV and gene expression data in context of regions with DA and equivalent accessibility. Single copy probe locations of regions with DA (in bold) and equivalent accessibility (i.e. without DA) are from indicated GRCh37 genomic coordinates. Overlapping CNVs in the population from Healthy Sample (HS) and Ontario Population Genomics Platform (OPGP) are separated by a “//.” No recurrent CNVs (diploid copy number state) were observed in regions overlapped by single copy probes, and are indicated by a “0”. A 179 kb gain in region of single copy probe within *C9orf66* was seen in only 1 out of ~400 HS individuals. This observation is a rare CNV and the region in which it resides, does not exhibit DA. SC probe locations do not overlap locations of CNVs reported in cell line GM06326 (see methods). GM10958 cell line CNV data have not been analyzed by genomic microarray analysis. This cell line has been characterized as phenotypically normal (Coriell Cell Repository). Compared to other tissues, mRNA abundance among human lymphocytes cells (EBV transformed, *n* = 54 individuals) showed low or no expression (most values below 0 on log10 scale, Genotype-Tissue Expression – GTEx database) for each of the single copy probes tested. *C9orf66* expression was not catalogued in GTEx. The EMBL expression atlas (Illumina body map, http://www.ebi.ac.uk/gxa/experiments/E-MTAB-513) confirmed that it is not expressed in leukocytes. Marks of transcriptionally active chromatin (i.e. H3K36me3, H4K20me1 - reported values represent integrated intensities) were not significantly present (*p* = 0.91) in either DA (in bold, *μ* = 170.86) or equivalent accessible genomic regions (*μ* = 161.15). Integrated intensity values were processed from ENCODE ChIP-seq data on lymphoblastoid cell line GM12878 using the Broad histone signal intensity ‘StdSig’ setting within the UCSC Genome Table browser. Additional file 9: Figure S5. Metaphase images of BAC FISH probes co-hybridized with scFISH probes. **(A)** BAC probe (RP11-57P14; green), which spans 187 kb on chromosome 9p21.2, consistently exhibits bright, equivalent hybridization signals on both homologs. Co-hybridized scFISH probe (red) is from within *CACNA1B* 9q34.3 treated with 0.25 μM (DA reversed). **(B)** Same BAC and scFISH probe as in **panel A** co-hybridized to metaphase chromosomes treated with 0.1 μM ICRF-193 (DA; asterisk indicates inaccessible homolog). **(C)** BAC probe (RP11-467L20; green) spanning 188 kb on chromosome 11q12.2 with bight signals to both homologs (derivative or ‘der’ 11 is a result of a translocation between chromosomes 1 and 11). Co-hybridized scFISH probe (red) is from within *RGS7* on 1q43 treated with 0.50 μM ICRF-193 (DA reversed). **(D)** Same BAC and scFISH probe as in **panel C** co-hybridized to metaphase chromosomes treated with 0.25 μM ICRF-193 (DA; asterisk indicates inaccessible homolog without *RGS7* hybridization). Additional file 10: Table S3. GVF measurements of BAC control FISH probe signal co-hybridized with scFISH probes. Columns labelled A and B represent integrated intensity measurements for each homolog in a diploid metaphase cell. Normalized integrated intensities were obtained by taking the difference in integrated intensities between homologs, and dividing by the sum of the intensities of both homologs in a given cell. Slides 1 (0.25 μM ICRF-193) and 2 (0.10 μM ICRF-193) were processed in parallel and co-hybridized with BAC control probe RP11-57P14 and scFISH probe *CACNA1B* with DA. Irrespective of whether ICRF-193 reversed DA (slide 1, 0.25 uM ICRF) or showed limited effect (slide 2), control probes hybridized with consistently bright intensities to both homologs. No significant differences (*p* = 0.85) were observed in normalized integrated intensities among control probes between slides 1 (Δμ = 0.147, Median = 0.156) and 2 (Δμ = 0.156, Median = 0.123). Slides 3 (0.50 uM ICRF) and 4 (0.25 uM ICRF) were processed in separate hybridizations and co-hybridized with BAC control probe RP11-467L20 and scFISH probe *RGS7* with DA. Similarly, no significant differences (*p* = 0.12) were observed in normalized integrated intensities among control probes between slides 3 (Δμ = 0.104, Median = 0.078) and 4 (Δμ = 0.162, Median = 0.110).

## Abbreviations

3D-SIM: 3-dimensional structured illumination microscopy
5-AZC: 5-azacytidine
H3K27me3: Histone H3 lysine 27 trimethylation
H3K36me3: Histone H3 lysine 36 trimethylation
H4K20me1: Histone H4 lysine 20 monomethylation
ChAS: Chromosome analysis suite
CNV: Copy number variation
DA: Differential accessibility
GVF: Gradient vector flow
ISCN: International system for human cytogenetic nomenclature
OA: Okadaic acid
POD-FISH: Parental origin determination fluorescence *in situ* hybridization
scFISH: Single copy fluorescence *in situ* hybridization
TSA: Trichostatin-A
