# Supplementary figures and images for: Reversing chromatin accessibility differences that distinguish homologous mitotic metaphase chromosomes
Source: Mol Cytogenet. 2015 Aug 13;8:65. doi: 10.1186/s13039-015-0159-y (PMC4535684; doi:10.1186/s13039-015-0159-y)

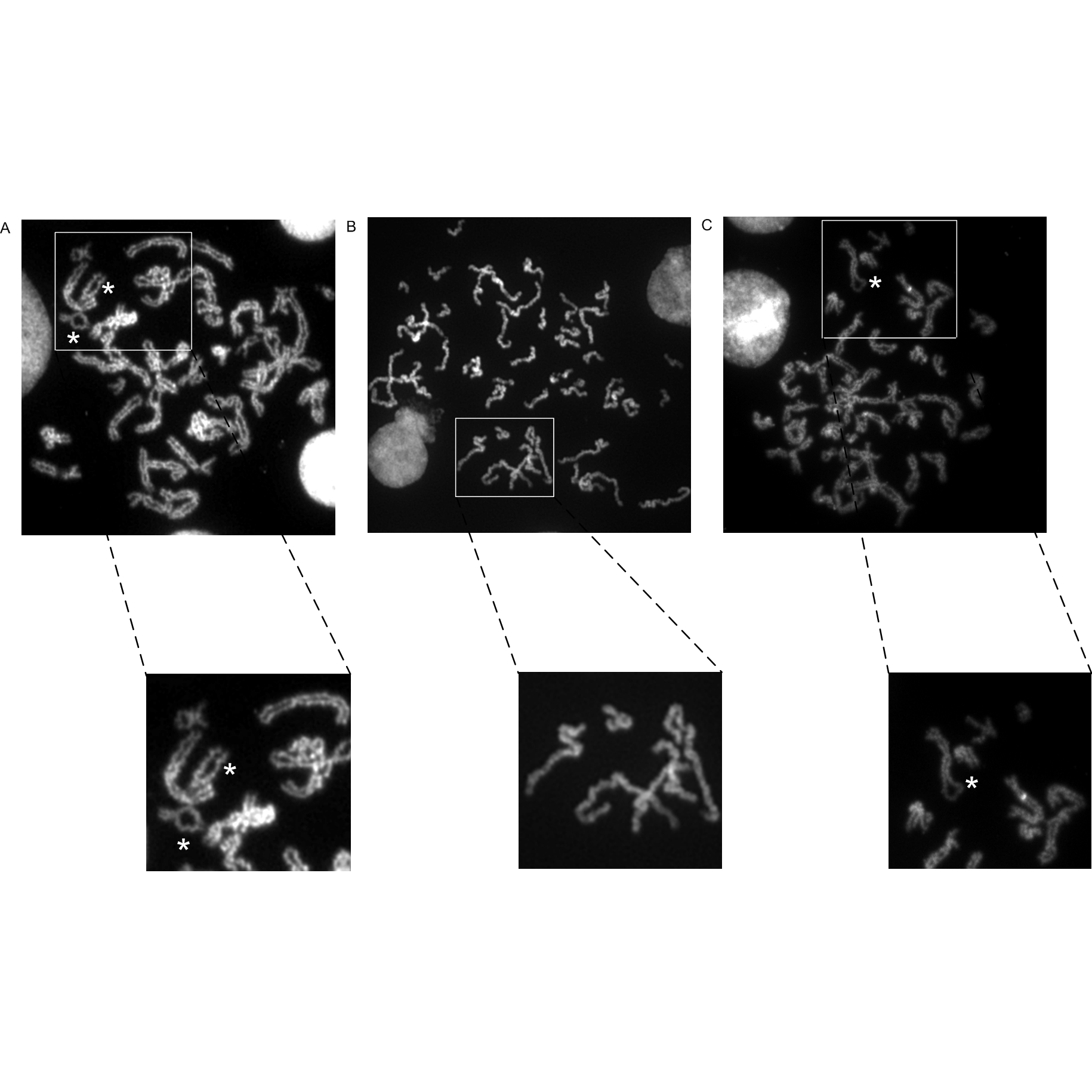

Supplement: Additional file 2: Figure S1. — White box indicates zoom in view of entangled or catenated metaphase chromosomes following (A) 0.25 μM and (B-C) 0.50 μM ICRF-193 treatment. Reduced supercoiling is visible as cytologically unwound chromatids (*). Panel B is same cell as shown in Fig. 1d. [file 13039_2015_159_MOESM2_ESM.tif]

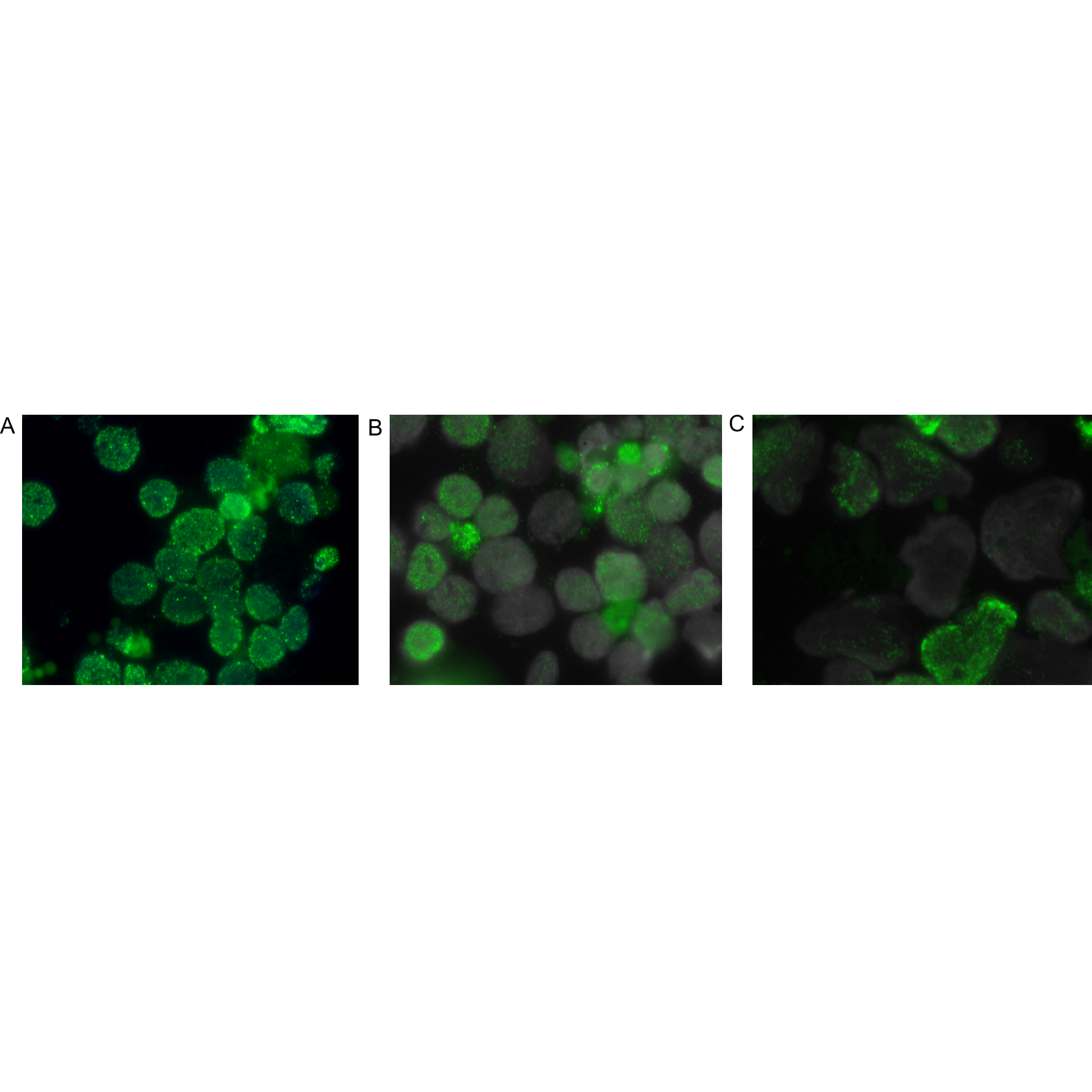

Supplement: Additional file 3: Figure S2. — Immunofluorescence staining of lymphoblastoid nuclei following selective inhibition of H3K27me3 associated with inactive chromatin. (A) H3K27me3 staining shows bright punctate nuclear signals in untreated cells, but diminished fluorescence and reduced signals post-treatment with (B) 5 μM and (C) 15 μM UNC1999, respectively. [file 13039_2015_159_MOESM3_ESM.tif]

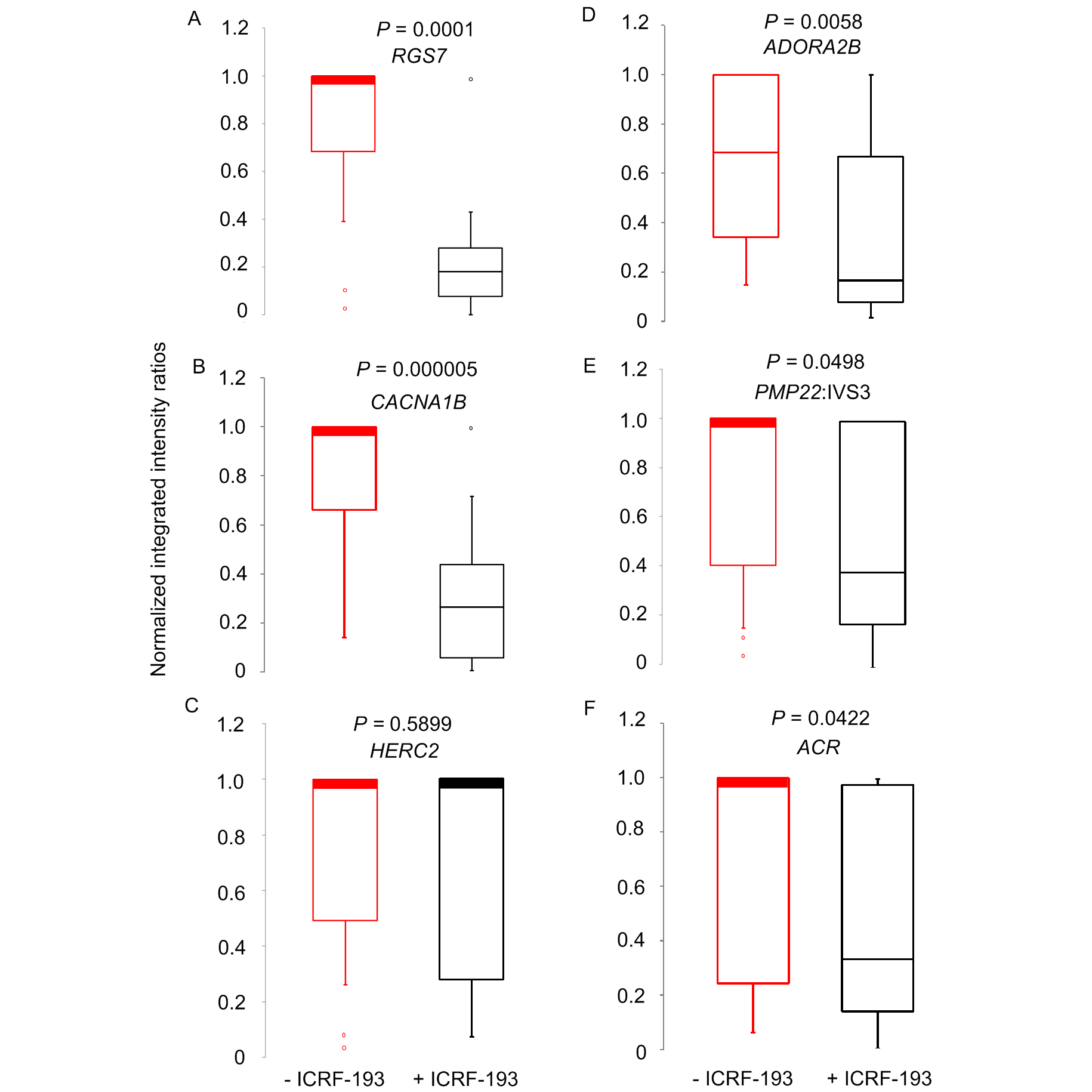

Supplement: Additional file 4: Figure S3. — Quantification of inter-homolog probefluorescence intensities following chromosome decondensation with ICRF-193 in independent cell lines. (A–F) Box plots show normalized integrated intensity ratios (y axis) following scFISH for six distinct genomic regions within chromosomes 1q43 (RGS7), 9q34.3 (CACNA1B), 15q13.1 (HERC2), 17p12 (ADORA2B, PMP22:IVS3), and 22q13.33 (ACR) in untreated (−) and treated (+) cells (x axis). GVF measurements in cells hybridized with single copy probes detecting DA from within RGS7, HERC2, PMP22:IVS3, and ACR are indicated from cell line GM10958. Measurements of normalized inter-homolog intensities for CACNA1B and ADORA2B are indicated from cell line GM06326. The same genomic regions were hybridized in opposite cell lines and inter-homolog differences quantified as shown in Fig. 3. Probes detecting DA exhibited larger differences in inter-homolog DNA probe fluorescence (red box plots: median intensity ratios: from 0.68 to 1, n = 125 cells). ICRF-193 treated chromosomes exhibited smaller differences in DNA probe fluorescence (black box plots: median intensity ratios from 0.15-0.39, n = 118 cells) (p < 0.05; two tail t-test), suggesting retrieval of the less accessible chromosome target, except in the case of HERC2, in which DA was not completely reversed. In instances where the median is coincident with the upper quartile, it is emphasized by a thick line to show distinction from the median in the corresponding category. [file 13039_2015_159_MOESM4_ESM.tif]

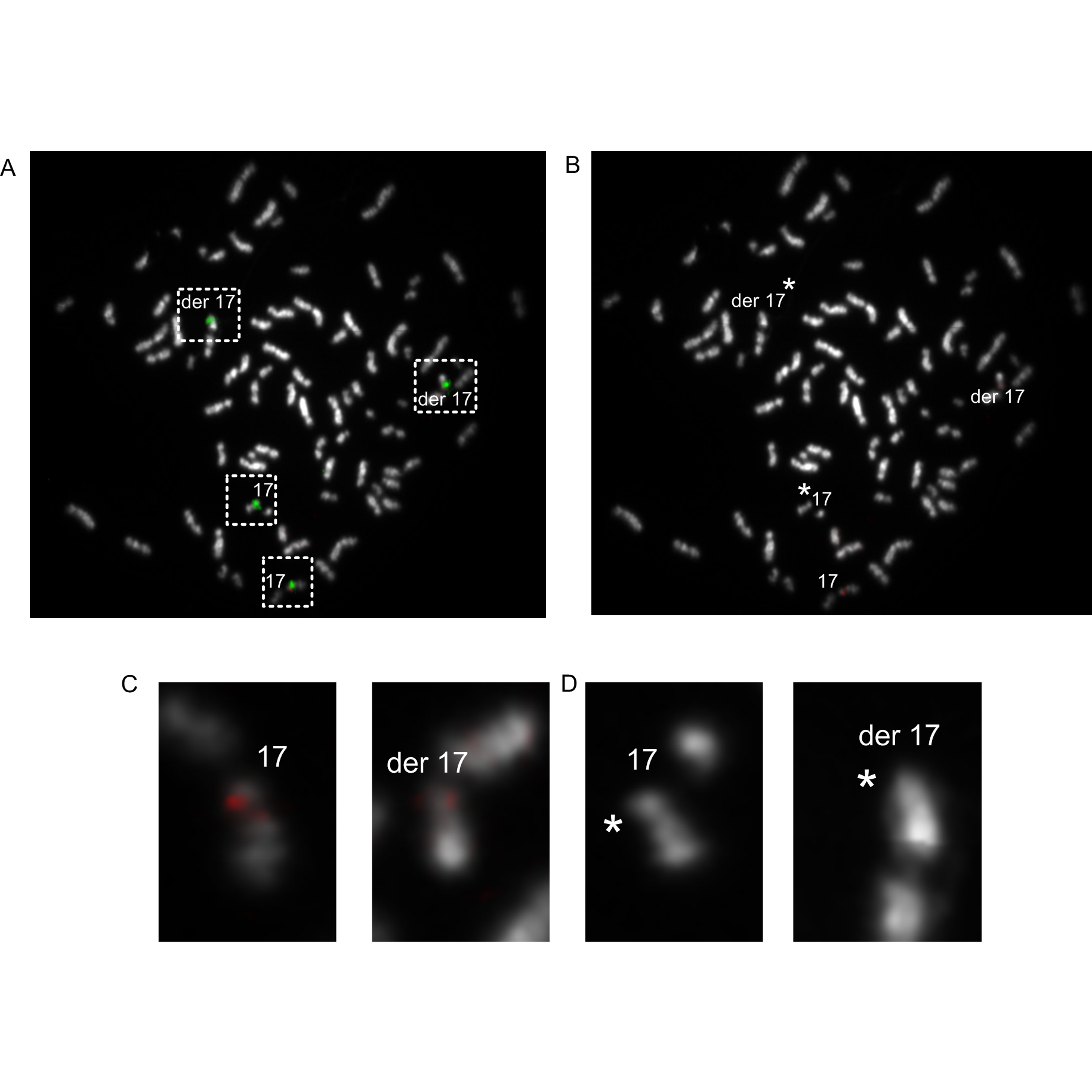

Supplement: Additional file 7: Figure S4. — Examples of DA in tetraploid-like cells after okadaic acid treatment. (A) Chromosome 17s are marked with centromeric probe (D17Z1, green) to identify the four copies in the tetraploid-like cell (boxed). (B) Tetraploid-like cell shows two of the four homologs hybridized with a 1.78 kb scFISH probe (red) within ADORA2B on chromosome 17p12, indicating DA. (C) Zoom in view of the same cell from panel B shows a bright hybridization to the normal chromosome 17 and a weaker hybridization to its corresponding homolog (observed in n = 16/25 cells). (D) The other pairs of normal chromosome 17 and der 17 (asterisk) showed absence of hybridization to their respective allelic targets (n = 12/28 cells). The same outcome shown in panel D was predominantly observed in a region with no DA (PMP22: IVS-Ex5) in which two of the four homolog pairs did not hybridize (n = 13/17 cells). Tetraploid-like cells with only single chromosome hybridizations, hybridizations to only two normal chromosome 17s or two der 17s were excluded, as they could not be analyzed for DA which is assessed between homologs. [file 13039_2015_159_MOESM7_ESM.tif]

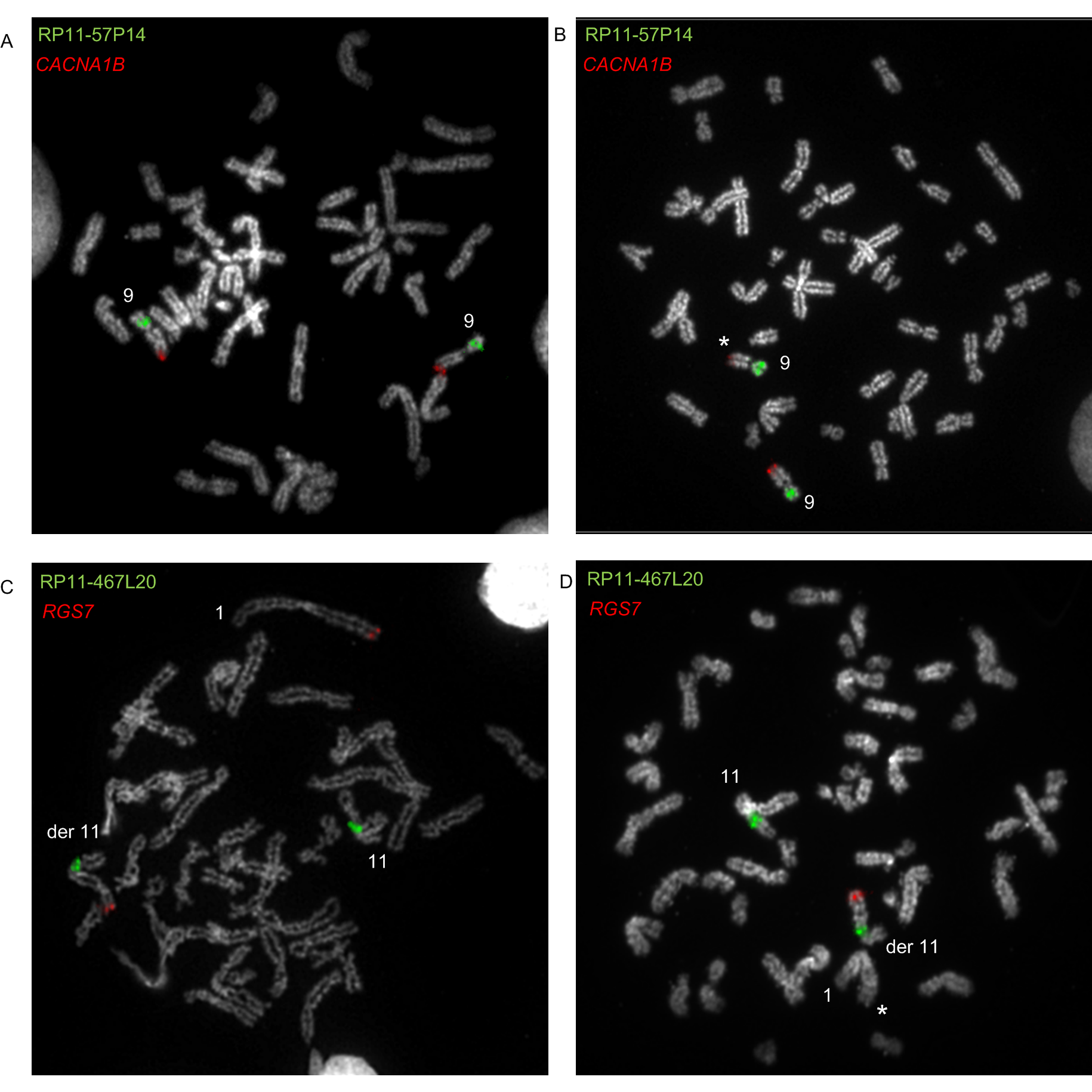

Supplement: Additional file 9: Figure S5. — Metaphase images of BAC FISH probes co-hybridized with scFISH probes. (A) BAC probe (RP11-57P14; green), which spans 187 kb on chromosome 9p21.2, consistently exhibits bright, equivalent hybridization signals on both homologs. Co-hybridized scFISH probe (red) is from within CACNA1B 9q34.3 treated with 0.25 μM (DA reversed). (B) Same BAC and scFISH probe as in panel A co-hybridized to metaphase chromosomes treated with 0.1 μM ICRF-193 (DA; asterisk indicates inaccessible homolog). (C) BAC probe (RP11-467L20; green) spanning 188 kb on chromosome 11q12.2 with bight signals to both homologs (derivative or ‘der’ 11 is a result of a translocation between chromosomes 1 and 11). Co-hybridized scFISH probe (red) is from within RGS7 on 1q43 treated with 0.50 μM ICRF-193 (DA reversed). (D) Same BAC and scFISH probe as in panel C co-hybridized to metaphase chromosomes treated with 0.25 μM ICRF-193 (DA; asterisk indicates inaccessible homolog without RGS7 hybridization). [file 13039_2015_159_MOESM9_ESM.tif]
